# Linking Transformational Leadership and Knowledge Sharing: The Mediating Roles of Perceived Team Goal Commitment and Perceived Team Identification

**DOI:** 10.3389/fpsyg.2018.01331

**Published:** 2018-07-31

**Authors:** Haixin Liu, Guiquan Li

**Affiliations:** ^1^Department of Information Management, School of Economics and Management, Beijing Jiaotong University, Beijing, China; ^2^Department of Human Resource Management, Business School, Nankai University, Tianjin, China

**Keywords:** transformational leadership, team goal commitment, team identification, knowledge sharing, leadership

## Abstract

It is widely assumed that transformational leadership can effectively facilitate the sharing of knowledge among followers, but most investigations of the underlying mechanisms were based on the social exchange perspective. Based on a sensegiving theory perspective, this article attempts to uncover the mechanisms by which transformational leadership has its impact on employee knowledge sharing behavior by proposing two team-directed mediating mechanisms: perceived team goal commitment and perceived team identification. Results of multi-source and time-lagged data from 186 leader–follower pairs supported the proposed mediating effects. Implications and limitations are discussed.

## Introduction

In today’s fast-changing business environment, a firm’s competitive advantage is highly dependent on its ability to generate and deploy new knowledge solutions (Grant, 1996). To this end, “knowledge sharing” is defined as the dissemination of ideas, information, expertise, and suggestions among individuals in organizations to solve problems, develop new ideas, or implement policies or procedures (Pulakos et al., 2003; Cummings, 2004; Wang and Noe, 2010). Highlighted by its definition as well as previous studies, knowledge sharing effectively promotes team collaboration (Wang et al., 2011), and triggers organizational change from as small as a revision of work policy to as significant as a new product design (Grant, 2013). Thus, it is crucial for leaders to facilitate knowledge sharing among followers. Over the past decade, an increasing number of scholars have emphasized the effects of various leadership styles on knowledge sharing (Bryant, 2003; Connelly and Kelloway, 2003; Lin and Lee, 2004; Srivastava et al., 2006; Yang, 2007, 2010; Nguyen and Mohamed, 2011; Xue et al., 2011; Li et al., 2014; Han et al., 2016; Masa’deh et al., 2016; Dong et al., 2017).

While all these studies have yielded interesting and useful insights, however, transformational leadership may be more important in promoting knowledge sharing at the individual level (Bryant, 2003; García-Morales et al., 2008). Consequently, considerable studies have reported transformational leadership’s positive impacts in various situations (Dong et al., 2017). Nevertheless, scholars begin to carefully consider whether facilitating knowledge sharing is as easy for leaders as simply exhibiting a number of transformational behaviors. Grounded in the social exchange theory, a large body of research then explored the underlying mechanism through which transformational leadership facilitates knowledge sharing. For example, most studies concluded that leader–member exchange is a crucial mediator that translates transformational leadership into individual knowledge sharing behavior (e.g., Li et al., 2014).

A social exchange theory perspective helped uncover the hidden mediating mechanisms of the transformational leadership knowledge sharing relationship on the one hand, but limited the investigation on other possible paths on the other hand. At its most basic, knowledge sharing typically takes place in a team context where team members share their ideas and experiences on work-related issues with each other (Hansen et al., 2005; Wang and Noe, 2010). Though knowledge sharing among team members will not necessarily benefit the individuals within the team, it will always benefit the team as a whole (Taylor, 2006). Thus, individual perceptions of team characteristics serve as important motivational determinants of individual knowledge sharing behavior. The crux of enhancing knowledge sharing, then, is to make team-related characteristics meaningful for team members.

Following this cue, we set out to examine how transformational leadership influence followers’ team-directed perceptions, which in turn impact followers’ knowledge sharing behavior from a sensegiving perspective that depicts leader’s attempts in influencing the meaning construction of followers toward a preferred redefinition of reality (Gioia and Chittipeddi, 1991; Maitlis and Lawrence, 2007). Specifically, apart from building high quality leader–member exchange relationships, a prominent function of transformational leaders is to generate meaningfulness-related outcomes such as “inspiring followers to transcend their own self-interests for a higher collective purpose, mission, or vision” (Howell and Avolio, 1993, p. 891). In other words, transformational leadership is particularly crucial in shaping the meaning of work for followers (Rosso et al., 2010). Thus, it is reasonable to believe that sensegiving is likely to be an important mechanism of the transformational leadership–knowledge sharing relationship.

Specifically, previous research asserted that leader sensegiving has two major consequences on the part of followers: one is to construct goal-related beliefs such as the meaningfulness of mission and purpose, while the other one is to frame identity-related issues (Podolny et al., 2005). Similarly, due to the aforementioned importance of team-related characteristics in determining knowledge sharing, we introduce two mediators, perceived team goal commitment and team identification, as two representing sensegiving mechanisms of the transformational leadership–knowledge sharing relationship.

Our study adds significant knowledge to the knowledge management literature. While many scholars have emphasized the importance of transformational leadership in motivating knowledge sharing (Li et al., 2014; Dong et al., 2017), our study demonstrates that one promising way that goes beyond the traditional social exchange perspective, is to shape their team-directed perceptions such as team goal commitment and team identification through the sensegiving process.

## Theory and Hypotheses

### Transformational Leadership and Knowledge Sharing

Transformational leadership inspires followers to go beyond their own self-interest and identify with the higher order vision and objectives (Bass, 1985). Over the past few decades, transformational leadership has been recognized as one of the most studied leadership topics. Recently, increasing debates on the dimensional structure of transformational leadership under different culture have attracted scholarly attention. There is support for a unidimensional construct (Berger et al., 2011), while others claimed for a four-factor structure (e.g., Bass and Avolio, 1995). Specifically, investigations in China showed that the four-factor model (MLQ, Bass et al., 2003; Wang et al., 2014), the five-factor model (MLQ5x, Bass and Avolio, 1995; Wu et al., 2010), and the six factor model (Podsakoff et al., 1990; Wang et al., 2005; Zhang et al., 2015) functioned satisfactorily. These results were reasonable and consistent with the conclusions of Global Leadership and Organizational Behavior Effectiveness (GLOBE) project that the US and Chinese cultures shared some similarities such as performance orientation, human orientation, and power distance, which reflect a preference for charismatic/value-based and team-oriented leadership in both cultures (Javidan et al., 2006). Thus, although deeply rooted in the Western culture, we believe that the concept of transformational leadership largely remains the same in both the US and Chinese cultures and is also prevalent in the Chinese culture. Thus, as most of the transformational leadership studies in the Chinese culture have repeatedly validated Podsakoff et al. (1990)’s transformational leadership model (e.g., Wang et al., 2005; Li et al., 2014; Zhang et al., 2015), we adopted the six-dimension transformational leadership in the current study.

More recently, however, according to different theoretical perspective, the dimensions of transformational leadership were grouped into different facets. For example, Wu et al. (2010) theorized transformational leadership as two sets of behaviors, namely, individual- and group-focused leadership behavior. In another vein, researchers have also identified the relationship- and task-focused dimension structure of transformational leadership (Wang et al., 2011). Despite of these inspiring findings, most studies tend to conceptualize transformational leadership as a unitary construct due to the high correlation between dimensions and lack of discriminant validity among dimensions (e.g., Hoffman et al., 2011; Epitropaki and Martin, 2013).

According to Podsakoff et al. (1990), it consists of six dimensions including articulating a vision, intellectual stimulation, high performance expectations, fostering collaboration, providing an appropriate role model, and providing individual support. Accumulated evidence has shown that transformational leadership positively associates with knowledge sharing (e.g., García-Morales et al., 2008). As Bryant (2003) argued, several dimensions of transformational leadership fit well with managing knowledge. For example, by articulating a challenging vision for the collective, encouraging intellectual development, and paying individualized attention, employees are motivated to create and share knowledge. Thus, as with many follow-up studies (Shao et al., 2012; Li et al., 2014; Le and Lei, 2018), transformational leaders effectively facilitate knowledge sharing in teams.

More studies continued to uncover the hidden mechanisms through which transformational leadership has its effect on knowledge sharing (Herman and Mitchell, 2010; Lee et al., 2010; Carmeli et al., 2011; Li et al., 2014; Han et al., 2016). To sum up, the majority of research focused on the exchange relationship between transformational leaders and employees based on social exchange theory (Lee et al., 2010; Li et al., 2014; Han et al., 2016). Specifically, Li et al. (2014) found transformational leadership positively influenced leader–member exchange, which in turn led to increased knowledge sharing. Other scholars also found transformational leadership facilitated knowledge sharing by enhance followers’ trust in leader (Lee et al., 2010). Although adopting a social exchange theory perspective has largely deepened the understanding on the underlying exchange processes, other theoretical perspective may also exist. Several studies have witnessed partial mediation effects of leader–member exchange and other similar constructs on the focal relationship (Li et al., 2014). Given that one prominent function of transformational leadership is shaping followers’ perception about team characteristics (Rosso et al., 2010), we hereinafter discuss how transformational leaders motivates knowledge sharing among employees via the sensegiving process.

### Transformational Leadership in a Sensegiving Perspective

Sensegiving refers to the attempts to influence others’ understandings of an issue (Gioia and Chittipeddi, 1991), which is embedded in the larger sensemaking process of an organization (Maitlis, 2005). Prior studies have identified typical sensegiving behaviors such as statements and activities that provide plausible description or explanations of extracted cues and construct sensible environment for others (Weick et al., 2005). Notably, leaders are the most important sensemakers who shapes followers’ perceived meaningfulness of work-related issues (Rosso et al., 2010).

According to previous studies, transformational leaders are typically associated with two sensegiving consequences (Podolny et al., 2005). The first one is the construction of employees’ goal-related beliefs, while the other one is the framing of employees’ identity-related issues. As aforementioned, knowledge sharing typically takes place in teams, and thus employees’ team-directed perceptions have the potential to motivate employees to share their knowledge. For example, employees’ team commitment associates positively with the intention of knowledge sharing (Liu et al., 2011). Therefore, we will discuss in a greater detail about how transformational leaders shape employees’ goal-related beliefs (i.e., perceived team goal commitment) and identity-related issues (perceived team identification). Given that there are insufficient evidence for differential effects of transformational leadership dimensions on sensegiving outcomes, in line with prior studies (Hoffman et al., 2011; Epitropaki and Martin, 2013), we conceptualize transformational leadership as one unitary construct.

### Making Sense of Team Goal

In work settings, a “team goal” generally refers to the task outcomes team members have to achieve (Weldon and Weingart, 1993). Numerous studies have shown that setting team goals is of great importance to improving team performance and effectiveness (e.g., Durham et al., 1997; Locke and Latham, 2002). Research has shown that the commitment of team members to team goals is also important in determining team outcomes in the Chinese context (e.g., Zhang and Chiu, 2012). While team members’ participation in the goal setting process facilitates the acceptance of team goals, leaders usually set the goals to give the team’s purpose and mission legitimacy (Aubé et al., 2014).

Transformational leaders define and articulate the team’s basic purpose and future directions and try to create a sense of excitement and vitality within the team (Wang and Noe, 2010). In this sensegiving process of team goal, for example, transformational leaders, as vision makers, arouse in followers a shared desire for specific needs and wants through vision articulation. Meanwhile, transformational leader also works as motivators and motivate followers to accept expanded responsibilities through intellectual stimulation and high performance expectations. Therefore, we argue that transformational leadership is particularly useful in making sense of team goals for followers, and thus increases their perceived commitment to team goal.

As the expectancy-value framework would suggest, a team member’s commitment to team goal largely depends on the expectancy that goal attainment is possible and the value placed on reaching the team goals (Weldon and Weingart, 1993). Thus, transformational leaders effectively increase follower’s perceived commitment to team goal in two ways. First, vision articulation increases follower’s perceived value of team goals. Second, high performance expectation and intellectual stimulation encourages follower to solve problems by using new and creative ways, strengthening his/her belief on team goals attainment. For example, Dionne et al. (2004) argued that while team leader’s visioning behavior could effectively facilitate team members’ the acceptance of shared team values, intellectual stimulation motivates team members to question ongoing assumptions and invent new uses of old processes. Shamir et al. (1993) demonstrated that leaders enhance follower’s efficacy about accomplishing a certain level of performance by expressing high expectations. Thus, it is reasonable to assume that task-oriented leadership relates positively to team member’s perceived commitment to team goals.

We also posit a positive relationship between perceived team goal commitment and knowledge sharing. Knowledge sharing is often seen as somewhat risky and, unless strongly motivated, individuals are not often willing to share their knowledge in order to avoid possible losses (Ipe, 2003). However, when a member is highly committed to team goals, he/she is more likely to direct his/her cognitive and behavioral resources toward attaining the team goals. For instance, when teams develop goals and values that members may accept, team members exhibit a higher level of extrarole behavior (Gregersen, 1993; Bishop et al., 2000), such as organizational citizenship behavior. In doing so, follower is both more likely to seek input from other team members and more receptive to team members’ advice and ideas. Thus, we come to the following hypothesis:

Hypothesis 1: Perceived team goal commitment mediates the relationship between transformational leadership and knowledge sharing.

### Making Sense of Team Identity

Team identification refers to the personal commitment and emotional involvement an individual has with a team. As social identity theory illustrates, members think themselves as part of the team and thus form a psychological connection through team identification. Therefore, perceived team identification captures the extent to which a work team is valued and contributes to a team member’s sense of self in the western culture (Ashforth and Mael, 1989) as well as in the Chinese culture (Tang et al., 2014).

Transformational leadership focuses on encouraging communication and teamwork to solve problems (Jiang and Chen, 2018). For example, transformational leaders directly advocate cooperation among team members by establishing a shared attitude, cultivating a helping climate, and asking team members to be “team players.” These behaviors cause followers to identify with the team. Behaving as an analyzer and a taskmaster, transformational leaders focus on making the team’s internal operating system more efficient by arousing employees’ affective responses, such as stronger perceived support and a higher commitment to organization (Wang and Noe, 2010). Thus, we argue that transformational leadership is also likely to make sense of team identities for employees.

A large number of studies show that perceived team identification is the product of leader behaviors such as consideration and benevolence (Cheng and Wang, 2015). Transformational leaders prioritize teamwork rather than individual work. Collaboration among teammates is encouraged. Thus, from an identity perspective, transformational leaders provide employees a sense of collective identity. Moreover, transformational leaders act as role models who offer help and support to others, cultivating a team climate where the team works together to solve problems. As the team leader serves as a symbol of the team, when the leader exhibits these supportive behaviors, other team members are likely to value this as a characteristic of the team, which may possibly elicit higher team identification.

In addition, a team member may have higher self-esteem because of the personal meaning and value that comes from belonging to a particular group (Bezrukova et al., 2009). A highly committed member contributes more personal resources for the team’s good (Bishop et al., 2000). For example, research has shown that individuals with a high level of team identification are more likely to show proactive work behaviors, such as organizational citizenship behavior (Gau et al., 2009). Thus, we argue that when other members are in need for help at work, members who identify with the team will proactively engage in disseminating knowledge to them, and possibly collecting knowledge from other members in return. Therefore, we propose the following:

Hypothesis 2: Perceived team identification mediates the relationship between transformational leadership and knowledge sharing.

## Materials and Methods

### Sample

Respondents include 186 leader–follower pairs from a large real estate corporation located in northwestern China. With the help of the human resources manager, all 196 team leaders participated the leader survey measuring demographic information and follower knowledge sharing behavior at two points in time. The team leaders were in charge of a variety of job functions, including marketing, sales, strategy, human resources, finance, and accounting. To form pairs, we obtained a roster of all employees and their team leaders with the help of the human resources manager and randomly selected one team member from each leader’s team.

We informed the participants there was no knowing or anticipated risks associated with the participation in this study, and that they may refuse to take part in the study or withdraw from the study at any time without jeopardizing their employment or any other rights before the survey. There were several measures taken to ensure confidentiality. First, we created a secret code for each questionnaire to match data, thus no identity information was required. Second, research assistants collected all the questionnaires immediately after completion, thus the team members were assured full anonymity; their leaders had no way of knowing their answers. Third, we told each respondent that the answers were to be used only for scientific research and were irrelevant to their work performance evaluation.

We administrated the survey three times with time lags of 2 weeks. At Time 1, we asked the leaders to fill out a questionnaire containing only demographic information. The randomly selected followers rated transformational leadership, as well as demographic information. Two weeks later, at Time 2, we asked the same followers to rate their commitment to team goals and team identification. Another 2 weeks later, at Time 3, we asked each leader to rate their follower’s knowledge sharing behavior.

All the respondents returned the questionnaires, and 186 of them were valid. Leaders had an average age of 38.27 years, and 128 (68.8%) were male. One hundred and five leaders (56.5%) hold college degrees: 62 (33.3%) have a bachelor’s degree, 16 have a master’s degree, and the remaining three have a doctorate degree. The average age of all 186 randomly selected followers was 29.71 years. One hundred and twenty-seven (68.3%) were male, and 47 (25.3%) have a college degree: 103 (55.4%) have a bachelor’s degree, 34 (18.3%) have a master’s degree, and the remaining two have a doctorate. We also asked how long each follower had worked with his/her team leader, and the result was 2.65 years (range = 1–13 years; *SD* = 1.68 years).

### Measurements

Except for knowledge sharing, which was rated by the team leaders, the study’s variables were measured by team members’ responses to questions using a 5-point Likert-type scale with answers ranging from “strongly disagree” to “strongly agree.” As we conducted the survey in a Chinese firm, we directly used the Chinese version of the transformational leadership measure that has been well validated in prior studies (e.g., Wang et al., 2005). For the rest variables, we used the translation-back-translation procedure suggested by Brislin (1980). First, we asked a management scholar who is a native English speaker and has a good mandarin skill to translate the original English scales into Chinese. Second, we asked a Chinese undergraduate student who knew little about management research to check the Chinese version with the purpose of identifying any grammatical errors and ensuring readability. Third, we asked another undergraduate student who majors in English translated the Chinese version into English. At last, the research team compared the two English scales and found satisfactory equivalency.

#### Transformational Leadership

We used a 23-item scale developed by Podsakoff et al. (1990) to measure transformational leadership. This scale has been used by prior studies in the Chinese culture and exhibited sufficient reliability and validity (Wang et al., 2005; Zhang et al., 2015). The six multi-item subscales corresponding to six dimensions include the following: (1) articulating a vision (sample question: “My supervisor inspires others with his/her plans for the future”); (2) intellectual stimulation (sample question: “My supervisor challenges me to think about old problems in new ways”); (3) high performance expectations (sample question: “My supervisor challenges me to set high goals for myself”); (4) fostering collaboration (sample: “My supervisor encourages subordinates to be team players”); (5) providing an appropriate role model (sample: “My supervisor leads by example”); and (6) providing individual support (sample: “My supervisor behaves in a manner thoughtful of my personal needs”; α = 0.77).

To further gauge the factorial structure of the transformational leadership construct, we conducted a set of confirmatory factor analyses. Results showed that the six-factor model with a higher order factor fitted data better [χ^2^ (*df =* 224) = 490.35, *p* < 0.001, Comparative Fit Index = 0.93, Tucker–Lewis Index = 0.92, root mean-square error of approximation = 0.07] than three alternative theoretical transformational leadership models including a one-factor model [Δχ^2^ (Δ*df =* 6) = 552.08; RMSEA = 0.14; CFI = 0.73; TLI = 0.70], a four-factor model [Δχ^2^ (Δ*df =* 2) = 300.15; RMSEA = 0.12; CFI = 0.81; TLI = 0.79], and a five-factor model [Δχ^2^ (Δ*df =* 1) = 165.45; RMSEA = 0.10; CFI = 0.84; TLI = 0.85].

#### Perceived Team Goal Commitment

We measured this variable using three items adopted from Aubé and Rousseau (2005). Followers were asked to enumerate to what extent they endorse each of the following descriptions: “I am committed to pursuing the team’s goal,” “I think it is important to reach the team’s goal,” and “I really care about achieving the team’s goal” (α = 0.71).

#### Perceived Team Identification

We assessed this variable using four items adopted from Van Der Vegt et al. (2003). Followers were asked to enumerate to what extent they endorse each of the following descriptions: “I strongly identify with the other members of my work team,” “I would like to continue working with my team,” “I dislike being a member of this work team” (reversed), and “I feel emotionally attached to this work team” (α = 0.81).

#### Knowledge Sharing

A 4-item scale was adopted from Lin (2007) and was used to measure individual’s knowledge sharing. We replaced the word “I” with the term “this employee.” Each direct leader was asked to rate their randomly selected subordinate on the following: whether the employee “shares knowledge with other colleagues more frequently,” “tries to share knowledge with other colleagues,” “always makes an effort to share knowledge with other colleagues,” and “shares knowledge with colleagues who ask” (α = 0.78).

#### Control Variables

Like in previous studies (e.g., Xue et al., 2011), we controlled for the demographics variables of both leaders and followers – including age, gender, and education background. Previous research showed that leaders’ demographics could influence followers’ perceptions of leaders and thus lead to distinct work attitudes (Green et al., 1996) while followers’ demographic diversity significantly relates to knowledge sharing (Wang and Noe, 2010). We also controlled for the amount of time of the leader–follower pairs had worked together, as leaders with longer tenure tend to be less effective (Kirkman et al., 2004). Age and time with a given leader were given in years; gender was designated by 1 for male and 2 for female; and educational background by 1 for college, 2 for bachelor’s degree, 3 for master’s degree, and 4 for Ph.D.

## Results

### Descriptive Statistics and Confirmatory Factor Analysis

**Table 1** presents the mean, standard deviation, correlation, and reliability coefficient for each variable in our study. We conducted the confirmatory factor analysis to assess the distinctiveness of each major variable. Due to the large number of estimated parameters, we used dimension scores for the transformational leadership constructs to reduce model complexity and generate more reliable and stable estimations (e.g., Wu et al., 2010). The four-factor model (transformational leadership, perceived team goal commitment, perceived team identification, and knowledge sharing) showed acceptable fit with data [χ^2^ (*df =* 113) = 261.38, *p* < 0.001, Comparative Fit Index = 0.92, Tucker–Lewis Index = 0.91, root mean-square error of approximation = 0.08].

**Table 1:** Mean, standard deviation, and correlation among each variable.

	Mean	*SD*	1	2	3	4	5	6	7	8	9	10	11
(1) Leader age	38.27	7.29											
(2) Leader gender	1.31	0.46	-0.19*										
(3) Leader education	1.55	0.72	0.12	-0.18*									
(4) Follower age	29.71	4.62	0.09	-0.04	-0.10								
(5) Follower gender	1.32	0.47	0.01	-0.04	0.02	0.04							
(6) Follower education	1.95	0.69	0.05	0.10	-0.19*	-0.03	0.01						
(7) Time with current leader	2.65	1.68	0.08	-0.01	-0.04	0.25**	0.07	-0.12					
(8) Transformational leadership	2.44	0.70	-0.08	0.01	0.18*	-0.10	-0.07	-0.20**	-0.11	(0.77)			
(9) Team identification	2.46	0.83	0.18*	-0.02	0.34**	-0.07	0.02	-0.27**	-0.16*	0.26**	(0.81)		
(10) Team goal commitment	2.43	0.83	-0.01	0.09	0.01	-0.05	0.09	-0.13	-0.05	0.27**	0.11	(0.71)	
(11) Knowledge sharing	2.33	0.71	0.08	0.03	0.13	0.02	-0.05	-0.04	-0.04	0.31**	0.23**	0.35**	(0.78)


*N = 186, internal consistency reliabilities are in parentheses on the diagonal. ^∗^p ≤ 0.05, ^∗∗^p ≤ 0.01 (two-tailed).*

### Hypothesis Testing

As the theoretical model has two mediators, we followed Hayes’(2013) suggestions on testing multi-mediator model and applied non-parametric estimation method (bootstrapping) via SPSS Macro PROCESS (Model 4, bootstrap samples = 5000) to estimate the mediation effects. Results showed that the indirect effect of transformational leadership on knowledge sharing via perceived team goal commitment is significant (*B* = 0.08, SE = 0.03, *p* < 0.001, 95% CI: [0.03, 0.15]). Meanwhile, the indirect effect of transformational leadership on knowledge sharing via perceived team identification is also significant (*B* = 0.03, SE = 0.02, *p* < 0.05, 95% CI: [0.01, 0.09]). Thus, hypotheses 1 and 2 were supported.

### Supplemental Analysis

Although researchers have suggested that transformational leadership dimensions should not be distinguished due to their high inter-correlations and lack of discriminant validity (Tepper and Percy, 1994; Yukl, 2013), others encouraged leadership scholars to investigate the dimensional influences of transformational leadership, given that different dimensions may have different effects on outcomes via different mediators (van Knippenberg and Sitkin, 2013; Deinert et al., 2015). Following this advice, we conducted additional analyses to further gauge the dimensional effects of transformational leadership on knowledge sharing, as well as the intermediating mechanisms. Specifically, we tested the effect of each one of the six dimensions on knowledge sharing via team goal commitment and team identification.

As **Table 2** shows, bootstrap analysis results demonstrated that three dimensions (i.e., articulating a vision, intellectual stimulation, and high performance expectations) positively relate to knowledge sharing only through team goal commitment. However, providing individual support facilitates knowledge sharing only through team identification, and fostering collaboration and providing an appropriate role model have their positive impacts on knowledge sharing via both paths.

**Table 2:** Indirect effects of transformational leadership dimensions on knowledge sharing.

Mediator	Effect	SE	LLCI	ULCI
**Articulating a vision → knowledge sharing**				
Perceived team goal commitment	0.05	0.02	0.01	0.10
Perceived team identification	0.01	0.01	-0.00	0.04
**Intellectual stimulation → knowledge sharing**				
Perceived team goal commitment	0.05	0.02	0.02	0.09
Perceived team identification	0.01	0.01	-0.00	0.04
**High performance expectations → knowledge sharing**				
Perceived team goal commitment	0.05	0.02	0.02	0.09
Perceived team identification	0.01	0.01	-0.01	0.03
**Fostering collaboration → knowledge sharing**				
Perceived team goal commitment	0.04	0.02	0.00	0.09
Perceived team identification	0.03	0.02	0.01	0.08
**Providing an appropriate role model → knowledge sharing**				
Perceived team goal commitment	0.05	0.02	0.02	0.10
Perceived team identification	0.03	0.02	0.00	0.08
**Providing individual support → knowledge sharing**				
Perceived team goal commitment	0.00	0.02	-0.04	0.04
Perceived team identification	0.03	0.02	0.00	0.07


*The effects of control variables are not shown.*

## Discussion

Although existing studies have already shown that transformational leadership is crucial in facilitating knowledge sharing among team members, our understanding on the underlying mechanism is largely limited. Despite the commonly used social exchange theory perspective, our study showed how transformational leadership influences followers’ knowledge sharing behavior via a sensegiving perspective. In keeping with our hypotheses, the results extend the available literature, unearthing the mediating roles of team goal commitment and team identification on the relationships of transformational leadership with knowledge sharing. The implications of our findings and the limitations of our research are discussed below.

### Theoretical Implications

Perhaps our study’s most prominent contribution is the introduction of two important team-directed mediating mechanisms into the transformational leadership–knowledge sharing research. Although it is understood that transformational leadership is effective in facilitating knowledge sharing (Chen and Barnes, 2006; Behery, 2008), prior research was founded upon the social exchange perspective. We believe that the process by which a transformational leader motivates knowledge sharing is more complicated than just simply exhibiting a set of behaviors. Our study suggests that perceived team goal commitment and perceived team identification function as two mediums in this process. According to our knowledge, this is the first study that systematically investigates the team-directed mechanisms by which transformational leadership impacts knowledge sharing; it thus sheds light on both leadership and knowledge sharing.

We also highlight the pivotal roles of team-related perceptions. Research has shown group-focused incentives facilitate knowledge sharing among team members (Taylor, 2006), illustrating that members’ perceptions of team characteristics are crucial in motivating knowledge sharing. Yet we are surprised that so few studies investigated the roles of perceived team goal commitment and perceived team identification in the knowledge sharing process, given that these two constructs have long been recognized as important determinants of team and individual outcomes (Ashforth and Mael, 1989; Weldon and Weingart, 1993). This is possibly because scholars have paid too much attention to the individual characteristics and organizational reward systems, neglecting the crucial role of teams. In fact, most of individual knowledge sharing behavior occurs within the team to which he/she belongs. Thus, the current study fills this gap by empirically describing the positive influences of perceived team goal commitment and perceived team identification on knowledge sharing.

In addition, we contribute to the literature on leadership by providing new evidence on the sensegiving process of transformational leadership. Importantly, our results demonstrated that transformational leadership behaviors have a strong impact on employees’ meaningfulness of team identity and team goal. We demonstrated that transformational leadership does have other functional mechanisms besides the social exchange effects. Meanwhile, as with other studies (e.g., Deinert et al., 2015), our additional analyses revealed that different transformational leadership dimensions have different effects on followers.

### Managerial Implications

Because knowledge sharing is a key step to the successful implementation of knowledge management systems, it is important to understand how leaders’ behaviors lead to a higher level of knowledge sharing among followers. Understanding the mediating mechanisms helps leaders identify the possible outcomes of different behavioral strategies, which, in turn, leads to better synchronization between their actions and followers’ expectations. Specifically, our analysis shows that articulating a vision, intellectual stimulation, and high performance expectations can contribute to the establishment followers’ commitment to team goals while fostering collaboration, providing appropriate role models, and providing individual support lead to more identification of the team. All of the above serves to increase followers’ knowledge sharing behavior.

In addition, organizations need to pay particular attention in cultivating employees’ commitment to their teams’ goals. As suggested by many scholars, not only the team leader but also the organization should help employees set clear and explicit goals, while focusing on providing employees with training opportunities to improve their professional skill. Meanwhile, intrateam collaboration should be encouraged by organizational policies and regulations, which helps building a collective team identity for employees.

### Limitations

Like any study, this one is not without limitations. First, though we collected multi-source and time-lagged data, our study is still cross-sectional. Despite the time sequencing influences, we cannot draw any causal inferences from the results. Recognizing this limitation, we strongly encourage future researchers to adopt longitudinal designs and utilize lab experiments to better gauge the causal relationships in our research model.

Second, given that we focused only on revealing individual mechanisms, there might be some effective group level variables pertaining to the “leadership–knowledge sharing” process that we did not take into account. Preliminary findings by Kirkman et al. (2009) and Wu et al. (2010) demonstrate that the leadership is flexible as to analysis level, comprising behaviors targeted at both groups and individuals. Thus, another interesting avenue for future research is to explore how team leadership affects group level variables and as a result motivates followers’ knowledge sharing. Similarly, there are other potential mediators that this study did not include; thus, we encourage future study to provide more comprehensive evidence on the mediating mechanisms.

Third, we theorized and measured team goal commitment and team identification as individual perceptions of the team, which is consistent with our research question at the individual level and the dyadic nature of the sample. However, a better way to capture these two constructs is to assess them at the team level, and we should have measured more employees in the same team. We expect future study to adopt a more appropriate research design and apply multilevel modeling in testing the proposed model more rigorously.

Finally, we administrated the survey to one Chinese firm. Although this research design may effectively isolate the confounding factors such as national or organizational culture on one hand, it also limits the applicability of our findings in other research contexts. For example, Chinese culture is characterized as high collectivism. Employees in Chinese organizations are possibly easier to form collective commitment and identification. Will the mediating roles of team goal commitment and team identification still hold in a high individualism culture such as the US? Future work in other cultures can help verify the generalizability of our findings.

## Conclusion

In this paper, we set out to uncover the underlying mechanisms by which transformational leadership impacts employees’ knowledge sharing through the sensegiving perspective. Using time-lagged, multi-source data, the results showed transformational leadership facilitates knowledge sharing among employees by enhancing followers’ perceived team goal commitment and perceived team identification.

## Ethics Statement

Since there is no ethic committee or other similar institutions in the authors’ university or government departments in China, the research team worked with the top management team of the sampled firm to go through all the survey procedures and ensured that all the procedures were in accordance with the ethical standards of the institutional and/or national research committee and with the 1964 Helsinki Declaration and its later amendments or comparable ethical standards with written informed consent from all subjects. The study was approved by the ethical review board of Nankai University.

## Author Contributions

HL and GL contributed equally to the research. They worked together to design the research, collect and analyze the data, write the manuscript up, and attend to the revisions.

## Conflict of Interest Statement

The authors declare that the research was conducted in the absence of any commercial or financial relationships that could be construed as a potential conflict of interest.
